# Breast milk-associated late-onset group B streptococcus sepsis in preterm triplets: A case report and literature review

**DOI:** 10.5339/qmj.2025.25

**Published:** 2025-01-27

**Authors:** Manar Saleh, Wesam Abuqura, Fouad Abounahia, Ashraf Gad

**Affiliations:** ^1^Neonatal Intensive Care Unit, Women's Wellness and Research Center, Hamad Medical Corporation, Doha, Qatar; ^2^Pediatric Department, Weill Cornell Medicine-Qatar, Doha, Qatar *Correspondence: Manar Saleh. Email: msaleh13@hamad.qa

**Keywords:** Cerebrospinal fluid, necrotizing enterocolitis, ventriculoperitoneal shunt, group B streptococcus, late-onset group B streptococcus, breast milk

## Abstract

**Background:**

Late-onset group B *streptococcus* (LOGBS) sepsis is a notable cause of morbidity and mortality in preterm neonates. While the vertical transmission of group B *streptococcus* (GBS) during delivery is well established, the potential role of breast milk in the transmission of LOGBS is not as clearly understood. This case report examines a unique instance of preterm triplets developing LOGBS sepsis following maternal GBS mastitis, with the aim of investigating the possible association between breast milk and LOGBS infection in preterm infants.

**Case presentation:**

A set of preterm male triplets born at 30 weeks of gestation were admitted to the neonatal intensive care unit. At two weeks of age, the infants showed clinical manifestations of LOGBS sepsis, including septicemia. Additionally, one of the triplets developed meningitis complicated by hydrocephalus, while another developed necrotizing enterocolitis (NEC). Concurrently, their mother was diagnosed with mastitis and her breast milk cultures tested positive for GBS. The triplets were treated with systemic antibiotics. However, triplet B subsequently required a ventriculoperitoneal shunt for hydrocephalus management, and triplet C underwent laparotomy for NEC treatment.

**Conclusion:**

The occurrence of LOGBS sepsis in these preterm triplets, coupled with maternal GBS mastitis and positive breast milk cultures, raises critical questions regarding breast milk as a possible route of transmission for LOGBS. Understanding this relationship is vital for improving clinical practice, particularly in the management of recurrent infections in this vulnerable population.

## Introduction

Group B streptococcus (GBS) is the leading cause of neonatal sepsis in developing countries, which leads to serious short- and long-term health problems, including meningitis, pneumonia, and delays in developmental milestones.^1–4^ Late-onset group B *streptococcus* (LOGBS) is a common cause of neonatal infections, including meningitis, bone, joint, and soft tissue infections.^5^ In 2020, GBS colonization was reported in approximately 20 million pregnant women, resulting in 162,000 cases of neonatal invasive infection, including 37,100 cases complicated by moderate to severe neurodevelopmental impairment. Similarly, another review reported a case fatality rate of 7%, which doubled in preterm babies.^6^

Data from the USA show that LOGBS disease occurs from 7 to 90 days after birth, with an incidence of 0.4 per 1,000 cases of late-onset sepsis.^7^ Unlike early-onset GBS disease, the incidence of LOGBS is not affected by intrapartum antibiotic prophylaxis.^8^ Risk factors for LOGBS include preterm birth, maternal colonization, multiple gestation, and young maternal age. Preterm infants are at particularly high risk for LOGBS due to their weak immune systems, as maternal immunoglobulins are transmitted to the fetus after 32 weeks. Other factors contributing to this increased risk in preterm babies include disturbed gut microbiota, formula feeding, and long hospital stays.^9^

The source of LOGBS infection is believed to be maternal through vertical or horizontal transmission, or through infant nasopharyngeal or gastrointestinal colonization. Environmental and nosocomial sources have also been reported.^1^ A prospective cohort study conducted in Italy involving 100 infants with LOGBS showed that 66% of mothers had positive GBS cultures at vaginal/rectal sites, either during antenatal screening or at the time of neonatal LOGBS diagnosis. However, the rates of maternal positive results were higher when mothers were tested after the neonatal diagnosis of LOGBS.^10^ Additionally, a meta-analysis found that the odds ratio of LOGBS increases by 2.67 in infants born to GBS-colonized mothers compared with infants born to non-colonized mothers.^11^

Breast milk plays a fundamental role in the development of an infant's immune system by providing essential nutrients, beneficial bacteria, and immunoglobulins.^12^ These components are critical for establishing a healthy intestinal microbiota and enhancing the infant's immune defenses against pathogens.^1^ However, some case reports have identified breast milk as a potential source of invasive GBS infection, although this relationship is not yet fully established. One possible mechanism is that the infant's nasopharyngeal mucosa becomes colonized with GBS as it passes through the birth canal. Subsequently, the bacteria can be transmitted to the maternal mammary ducts during breastfeeding, leading to the potential for GBS to be present in breast milk.^1.^ We report a case involving a set of male triplets born preterm who developed LOGBS sepsis upon admission to the neonatal intensive care unit, at the Women's Wellness and Research Center (WWRC) in Qatar. Written consent was obtained from the legal guardian. The study was approved by the Institutional Board Review of Medical Research Center at Hamad Medical Corporation (approval number: MRC-04-23-754).

## Case Presentation

The mother, a healthy 30-year-old, was conceived through intrauterine insemination. She underwent a cesarean section at 30 weeks of gestation due to category II fetal heart rate tracing and premature labor. In particular, no prenatal GBS screening was performed. At birth, all three infants were vigorous. Triplet A had Apgar scores of 3 at 1 minute, 6 at 5 minutes, and 7 at 10 minutes. Triplet B had an Apgar score of 7 at both 1 and 5 minutes. Triplet C had Apgar scores of 6 at 1 minute, 6 at 5 minutes, and 9 at 10 minutes (Table 1). Growth parameters at birth for Triplet A were a birth weight of 1.56 kg, a height of 41 cm, and a head circumference of 31 cm. Triplet B had a weight of 1.45 kg, a height of 37 cm, and a head circumference of 29 cm. Triplet C had a weight of 1.35 kg, a height of 40 cm, and a head circumference of 27 cm.

Noninvasive ventilation was initiated in each infant immediately after birth, with sepsis screening and first-line empirical antibiotic therapy (ampicillin and amikacin) administered, and discontinued at 48 hours when blood cultures were negative. During the triplets’ hospitalization, at 18 days of life, the mother developed a fever and mastitis, for which she was treated with cloxacillin, while the babies continued to be fed exclusively with expressed breast milk (EBM).

## Clinical Course

### Triplet A

On day 14 of life, the baby was found to be bradycardic and hypoactive, the skin was mottled, and his blood gas showed metabolic acidosis. He was intubated and started on amikacin and teicoplanin according to hospital guidelines for presumed late-onset sepsis. The blood culture returned positive for GBS, so the antibiotics were immediately changed to penicillin G, which was administered for two weeks. Follow-up blood cultures were negative. Triplet A was discharged on day 45 of life (Table 1).

### Triplet B

**Figure 1.**
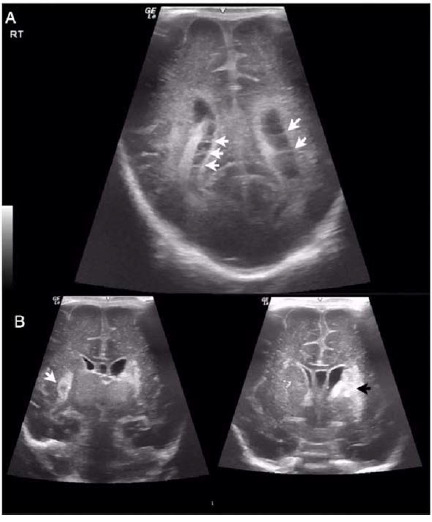
Ultrasonography images of the brain of triplet B following GBS sepsis at different time points. (A) Image taken on day 17 of life showing signs of ventriculitis characterized by thin septations (white arrowheads) and debris in the lateral ventricles, and turbidity in the cerebrospinal fluid. (B) Image taken at 1 month of age showing the development of bilateral parenchymal hemorrhages: a 2.3 × 2.0 cm hematoma in the left frontal cerebral region (black arrowhead) and a 1.8 × 1.3 cm hematoma in the right parietal periventricular area (white arrowhead).

**Figure 2.**
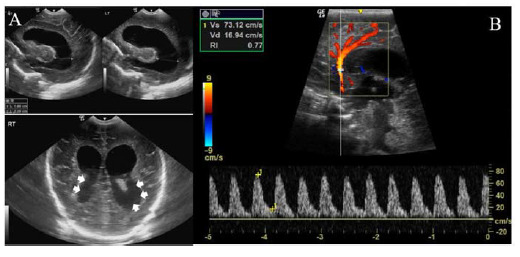
Brain ultrasonography of triplet B at six weeks. (A) Notable hydrocephalus is shown. Measurements include a diagonal diameter of 18 mm in the right frontal horn and 25 mm in the left frontal horn, yielding a ventricle-to-cranium ratio of 4.3:8.6, equating to 50%. Additionally, the cerebrospinal fluid shows turbidity in the occipital horn of the lateral ventricle (white arrowheads). (B) Pulsed wave Doppler results are shown, indicating an increased resistive index (RI) of 0.77, surpassing the 90th percentile for this age group, suggestive of increased vascular resistance secondary to hydrocephalus.

On day 15, he was diagnosed with GBS septicemia and meningitis, as evidenced by abnormal cerebrospinal fluid (CSF) profiles and a peak C-reactive protein (CRP) level of 183 mg/L (Table 1). An initial ultrasound examination of the head two days later showed thin septations and debris in the lateral ventricles, with turbid CSF indicating ventriculitis, no midline shift, and no hemorrhage (Figure 1). A follow-up brain ultrasound at one month of age revealed bilateral cerebral hematomas (Figure 2). A brain magnetic resonance imaging performed on day 35 revealed changes in the basal ganglia and left frontal and right frontoparietal paraventricular subacute to early chronic changes with leptomeningitis. A repeat brain ultrasound scan at six weeks of age revealed severe hydrocephalus, requiring placement of a ventriculoperitoneal shunt. Triplet B received penicillin G from day 15 of life and completed a total of six weeks as recommended by the infectious disease team.

### Triplet C

Although triplet C was initially asymptomatic and on full EBM feeding, he was screened and given prophylactic penicillin G due to the history of his siblings. Antibiotics were discontinued on day 5 after two negative cultures and normal CRP levels. However, on day 24, four days after stopping antibiotics, he showed signs of sepsis and lactic acidosis, requiring mechanical ventilation for one day. His blood culture returned positive for GBS, but CSF and urine cultures were negative (Table 1). On the ninth day of antibiotic treatment, the baby developed abdominal distention and bloody gastric aspirates, leading to the diagnosis of necrotizing enterocolitis. The baby underwent a laparotomy, during which 3 cm of the small intestine was removed due to ileal bowel necrosis and a stoma was created. The stoma was subsequently closed at 2.5 months of age.

Given the maternal history of mastitis and subsequent infection in the triplets, an investigation of EBM on day 26 of life revealed a positive result for GBS (colony-forming unit < 10^4^) and methicillin-sensitive *Staphylococcus aureus*. EBM was temporarily withheld and all babies were started on formula feeding. The mother received cloxacillin for a total of seven days. EBM culture was not repeated and all babies resumed EBM feeding on day 39 of life until hospital discharge.

## Discussion

Breast milk has been posited as a potential factor in the occurrence of LOGBS infections, as suggested by several case reports.^13–15^ The proposed mechanism involves the translocation of bacteria from the gut mucosa to the bloodstream, sometimes in association with maternal mastitis. It has been hypothesized that GBS could be transmitted to the mammary glands through the gut via lymphatics.^1^

Studies suggest that between 0.5% and 3% of mothers carry GBS in their breast milk, potentially posing a risk for neonatal infection.^16^ In our cases, maternal mastitis represented a significant risk factor for the presence of GBS in breast milk. These neonates had several risk factors that increased their susceptibility to invasive GBS infection. Preterm infants have underdeveloped and less coordinated immune systems, making them particularly vulnerable to infections.^17^ Their susceptibility is further compounded by factors such as disturbed gut microbiota, which is common in preterm infants due to antibiotic use and lack of exposure to maternal vaginal flora.

There is an increased risk of invasive GBS infection in multiple births, and breast milk feeding is implicated in several cases.^13–15^ In a case report involving multiple children in a Chinese family, the same GBS serotype III CC17 found in the mother's milk was isolated from a set of dizygotic twins and a pair of siblings.^13^ Similarly, a serious case of breast milk-associated transmission of recurrent LOGBS sepsis and meningitis in twins was also reported, with one twin succumbing to meningitis.^14^ In another report, the GBS pathogen was identified as the same serotype III in both twins.^15^ According to a meta-analysis, a twin of an infected infant may be at a 25-fold increased risk of infection.^11^

Notably, cases of recurrent infections associated with GBS-contaminated breast milk have been documented at 25% for two episodes and 7% for three episodes. Some authors have even suggested conducting breast milk cultures for recurrent infections.^2^ Another meta-analysis that included nearly 5,000 infants reported odds ratio for GBS infection of 19.7 for preterm births, 8.1 for twins, and 6.73 for low birth weight infants, highlighting that preterm babies and those with very low birth weight are at particularly high risk for invasive GBS infection.^18^

Our case demonstrates the horizontal transmission of GBS via breast milk associated with maternal mastitis leading to LOGBS sepsis. A case reported in 2022 by Zhejiang University in China involved a preterm neonate born at 32+2 weeks who developed LOGBS at 20 days of age. The CSF culture of the neonate yielded negative results. The mother tested positive for GBS before delivery and received treatment. However, she developed fever and mastitis when the baby was 9 days old. After LOGBS, both cervical and breast milk cultures were collected from the mother, and whole-genome sequence analysis was performed on maternal and neonatal sources. The analysis showed a 100% homology between breast milk and neonatal culture, while cervical culture showed a different result.^19^

In another case from Japan, a term infant was admitted with GBS meningitis and septicemia. The infant's mother had mastitis and cultures showed the same serotype and strain in both the infant's pharyngeal swab and maternal breast milk.^20^ Therefore, understanding these types of transmission helps identify and implement appropriate preventive and management strategies for infectious diseases.

A case reported by Ager et al. documented preterm twins born at 30 weeks. Twin A suffered from LOGBS on day 33 of life, followed by twin B. Both twins tested positive for GBS in their blood cultures. The breast milk and breast pump underwent testing, yielding positive GBS results. Pulsed gel electrophoresis and whole-genome sequencing confirmed the identical isolates from both breast milk and blood.^21^

Contrary to existing evidence linking breast milk to LOGBS in the presence of mastitis, not all infants who consume culture-positive breast milk develop infections. This is supported by reports that found no association between LOGBS and preterm infants fed culture-positive EBM from mothers with clinical GBS mastitis.^22,23^ Additionally, a recent case–control study in Australia concluded that breast milk is not directly associated with LOGBS. However, the study found a significant relationship between LOGBS and maternal positive GBS screening.^24^

The association between breast milk and its role in LOGBS remains a subject of controversy. It is important to note that the majority of breastfed infants do not develop GBS infections, and that breast milk plays a critical role in developing the immune system as it is a rich source of anti-GBS antibodies, particularly in preterm infants.^16^ Therefore, this association could be case-specific, especially when there is a high suspicion of maternal mastitis. In such cases, careful monitoring is necessary, particularly in preterm neonates. Further objective studies and meta-analyses are needed to clarify the relationship between breast milk contamination and GBS in neonates, especially in recurrent cases.

## Conclusion

This case report describes a set of preterm triplets who developed LOGBS sepsis associated with maternal mastitis caused by the same organism. The infection was further complicated by meningitis and hydrocephalus in one triplet and NEC in another. This report highlights the complex relationship between breast milk and LOGBS infections in neonates. Although breast milk is essential for the nutrition and immunological protection of preterm infants, it can also serve as a potential route of transmission for GBS in cases of maternal mastitis. A balanced approach is necessary to consider both the benefits and risks of breastfeeding when GBS is present. Further research is needed to understand the role of breast milk in GBS transmission and to develop safer breastfeeding practices when there is an increased risk of maternal GBS infection.

### Ethical statement

The study was approved by the WWRC Medical Research Center under protocol number MRC-04-23-754. Informed consent was obtained.

### Competing interests

The authors have no conflicts of interest to declare.

### Authors’ contributions

**Manar Saleh** drafted the initial manuscript. **Wesam Abuqura** contributed to the clinical data. **Fouad Abounahia** reviewed the manuscript. **Ashraf Gad** provided graphical data, and edited and reviewed the manuscript.

## Figures and Tables

**Table 1:** Comparative summary of demographics, clinical and laboratory characteristics, management, and outcomes in triplets.

	Triplet A	Triplet B	Triplet C

Birth weight (g)	1,560	1,450	1,350

Gender	Male	Male	Male

APGAR scores at 1,5, and 10 minutes	3,6, 9	7,7	6,6, 9

Initial NICU diagnosis	RDS	RDS	RDS

Onset of symptoms	Day 14	Day 15	Day 21

Maternal symptoms	The mother developed fever and mastitis on day 18 and underwent EBM culture on day 26. She received a 7-day course of cloxacillin from day 23.		

Clinical manifestations	Respiratory distress, tachycardia, poor perfusion	Bradycardia, poor perfusion, mottled skin, lethargy, with episodes of apnea	Bradycardia, desaturations, hypoactivity

Respiratory support	Upgraded from CPAP to NIPPV	Upgraded from CPAP to conventional mechanical ventilation for 4 days	Upgraded from CPAP to conventional mechanical ventilation for 1 day

Blood culture	Day 15: GBS Day 17: no growth	Day 15: GBS Day 18: no growth	Day 15: no growth Day 24: GBS Day 27: no growth

CSF culture	Day 15: no growth	Day 16: GBS Day 19: GBS Day 27: no growth	Day 26: no growth

Urine culture	Day 15: no growth	Day 16: no growth	Not done

CRP (mg/l)	Day 15: 55 Day 17: 11	Day 15: 111 Day 17: 183 Day 18: 126 Day 23: 16	Day 24: < 5 Day 25: 82 Day 31: 17

Other (during GBS sepsis)	Hyperglycemia, metabolic and respiratory acidosis, and lactic acidosis	Hyponatremia, hyperglycemia, metabolic and respiratory acidosis, lactic acidosis, and elevated liver enzymes	Metabolic acidosis Lactate acidosis

Platelet count ( × 10^3^/μl)	620	240	586

White blood count ( × 10^3^/μl)	22	34.3	30

CSF parameters	Normal chemistry and cell count	CSF white cell count (counts/μl): Day 16: 40 Day 19: 83 Day 27: 657 CSF RBC (counts/μl): 1,167>41,000>6 CSF protein (g/l): 3.36>4.14>3.33 CSF glucose (mmol/l): 0.28>1.63>1.15 Serum glucose (mmol/l): 6.9>4.8>5	Normal chemistry and cell count

Antimicrobials received	Day 14 of life: Penicillin G for 16 days, amikacin for 5 days	Day 15 of life: Penicillin G for 6 weeks, amikacin for the first 5 days	Day 15 of life: Penicillin G for 5 days (negative culture) Day 24 of life: Penicillin G for 9 days Day 32 of life: NEC treatment (meropenem)

Outcome	Bacteremia resolved, discharged home at day 45 of life	Meningitis, ventriculitis, intraparenchymal brain hemorrhage, obstructive hydrocephaly requiring VP shunt	Bacteremia resolved, NEC surgical


NICU, neonatal intensive care unit; RDS, respiratory distress syndrome; EBM, expressed breast milk; CPAP, continuous positive airway pressure; NIPPV, nasal intermittent positive pressure ventilation; GBS, group B *streptococcus*; CSF, cerebrospinal fluid; NEC, necrotizing enterocolitis; VP shunt, ventriculoperitoneal shunt.
